# Editorial: Advances in dental pulp stem cell biology and applications

**DOI:** 10.3389/fcell.2025.1695340

**Published:** 2025-09-26

**Authors:** Simona Delle Monache, Fanny Pulcini, Anne George

**Affiliations:** ^1^ Department of Biotechnological and Applied Clinical Sciences, University of L’Aquila, L’Aquila, Italy; ^2^ Department of Oral Biology, University of Illinois Chicago, Chicago, IL, United States

**Keywords:** mesenchymal stromal cells, CD146, dental pulp stem cells, odontogenic regeneration, odontoblastic differentiation, DPSCs immunomodulation, DPSCs

Dental pulp stem cells (DPSCs) are a type of mesenchymal stromal/stem cell (MSCs) found in the tooth’s innermost layer. Discovered in 2000, they have gained attention for their high proliferation rate, strong differentiation potential, and ease of access (Dominici et al., 2006). DPSCs have shown promise in regenerative medicine applications like dental tissue regeneration, cardiac and bone repair. Because their origin from ectomesenchyme, also have neurogenic capabilities, suggesting their potential use in neurodegenerative diseases. Therefore, due to the restorative properties of DPSCs, as well as their immunomodulatory, anti-inflammatory, pro-angiogenic, and tropic abilities, considerable efforts have been made to introduce advanced MSC-based therapy into clinical practice. Current research is now centered on unravelling the molecular and cellular mechanisms that govern their actions, as well as exploring the role of external factors and extracellular matrix components in DPSCs fate and the applications of DPSCs in tissue regeneration, also using specific scaffolds and delivery systems for enhancing DPSC-based tissue engineering. This Research Topic summarizes recent developments in this field and looks at the relationship between fundamental biology and novel therapeutic approaches.

One of the biggest hurdles in using mesenchymal stromal cells (MSCs) for clinical therapies is the inconsistency of results in clinical trials. This is primarily due to the natural heterogeneity of MSCs, which varies based on the donor, tissue source, and the cells’ current state (Garcia-Ber et al., 2021). Furthermore, differences in how the cells are isolated, cultured, and expanded can alter their function (Galipeau and Sensebe, 2018; Mattei et al., 2025). A study by Milek et al. on periodontal ligament MSCs (PDL-MSCs) highlighted the importance of using specific markers, like CD146 to isolate subpopulations with superior proliferation and osteogenic differentiation potential. Researchers observed that critical variables such as cellular density or confluence can negatively affect the percentage of CD146+ cells, thereby impacting cellular potential. They also found that CD146 expression can be influenced by inflammatory cytokines such as IL-1β and TNF-α. Although this study was on PDL-MSCs, CD146 could be considered a reliable marker for identifying a subpopulation with superior osteogenic differentiation potential. Therefore, a key area for future research in this field is the identification of specific markers to isolate more powerful subpopulations for use in regenerative therapies.

Researchers are also looking into how epigenetics plays a part in how cells change. A thorough review highlighted that key epigenetic mechanisms like DNA methylation, histone modifications and non-coding RNAs (miRNAs and lncRNAs) act as molecular switches that control the differentiation of DPSCs (Huang et al.). For instance, DNA demethylation and histone acetylation activate essential dentin formation genes (DSPP and DMP1), suggesting the potential for developing targeted therapies that can actively modulate these mechanisms to enhance regeneration. Moreover, this review explores the potential involvement of histone modifications, particularly methylation and acetylation, in regulating the chromatin structure and gene expression that drives the differentiation of DPSCs reporting how histone acetylation, catalysed by enzymes such as p300, can relax chromatin structure and activate essential differentiation genes. It has also been documented that HDAC inhibitors (HDACis) can affect gene expression by regulating histone acetylation levels, thereby influencing the differentiation and proliferation of DPSCs. This shows great promise for pulp regeneration (Liu et al., 2015; Sultana et al., 2021; Paino et al., 2014). Regarding the function of non-coding RNAs (ncRNAs), such as microRNAs (miRNAs) and long non-coding RNAs (lncRNAs), it has been reported that the lncRNA ANCR inhibits odontogenic differentiation, whereas the lncRNA MALAT1 promotes it (Chen et al., 2016; Bao et al., 2020; Huang et al., 2012). This review is of fundamental importance because it shifts the focus from simply cultivating and inducing DPSCs to developing a deeper understanding of the molecular mechanisms that regulate their behavior. The epigenetic approach offers new ways to improve the effectiveness of regenerative therapies, such as developing targeted therapies based on methylation inhibitors.

Also, by studying the signaling pathways that govern the differentiation mechanisms of DPSCs, Kim et al., for example, identified a new signaling mechanism that operates in an inflammatory environment, demonstrating that inhibition of the CaMKII pathway enhances the odontogenic differentiation of hDPSCs via the TrkB receptor, even in the presence of TNF-α-induced inflammation. This finding is of vital clinical importance as it suggests that regenerative processes can be promoted under adverse conditions, which is fundamental to the success of dental tissue engineering strategies.

In addition to understanding the biological mechanisms that regulate DPSC behavior, research is focusing on enhancing their effectiveness *in vivo*, particularly by stimulating vascularization, which is essential for the survival and integration of any graft. A recent study has shown that it is possible to induce the differentiation of DPSCs into functional endothelial cells (ECs) (Ganapathy et al.). The research used DMP1 (dentin matrix protein 1) and a HUVEC extracellular matrix scaffold to stimulate this differentiation, demonstrating that the CD31+/CD144+ fraction retains both the phenotypic and functional characteristics of ECs, in contrast to the CD31−/CD144− fraction (Ganapathy et al.). A targeted approach of this kind could overcome one of the most significant challenges for the regeneration of complex tissues such as dental pulp.

Another promising strategy focuses on the optimization of the regenerative microenvironment. One study proposed the use of nucleus pulposus microspheres (NPMs) as a biodegradable scaffold to deliver conditioned medium (CM), a rich mixture of growth factors that DPSCs secrete. The researchers measured the expression of the key genes DSPP and DMP-1, as well as alkaline phosphatase (ALP) activity and mineralized nodule formation, in order to evaluate the differentiation of cells into those that form dentin (Kim et al.). They then assessed pulp regeneration by implanting the DPSC + NPM + CM complex in a mouse model. This approach exploits the paracrine effect of stem cells, demonstrating that odontogenic differentiation and angiogenesis can be promoted without the need for large-scale cell transplantation. The effectiveness of this approach in completely regenerating pulp tissue in animal models paves the way for cell-free or low-cell-load therapies.

Furthermore, it should be emphasized that the effectiveness of DPSCs depends on the context in which they are used. A fascinating study by Pisciotta et al. revealed that, in a fibro-inflammatory microenvironment, DPSCs can induce an anti-fibrotic transition in the early stages, but they risk acquiring a pro-fibrotic phenotype if the inflammation becomes chronic. This transition appears to be mediated by bone morphogenetic protein (BMP2)-dependent pathways. The study also showed that DPSCs can modulate the expression of markers such as α-SMA, fibronectin and collagen, and/or PPARγ, by inhibiting the pro-fibrotic effects of TGF-β1. This discovery is crucial for clinical practice as it helps to understand the therapeutic potential of hDPSCs, especially in clinical contexts characterised by inflammation and fibrosis, such as autoimmune diseases and tissue regeneration.

In conclusion, stem cell research is progressing from the discovery phase to molecular and cellular engineering. DPSCs are a promising resource with the potential to become an effective therapeutic tool. However, to realize this potential, we must improve our understanding of how cells behave in an inflammatory environment and of the mechanisms involved in epigenetic and signalling processes.
